# New Test for Alcohol

**Published:** 1872-04

**Authors:** 


NEW TEST FOR ALCOHOL.—Berthelot observes that benzoyle chloride $C_{14}H_5ClO_2$ is not readily decomposed by cold or luke-warm water; if, however, alcohol is present, benzoic ether is at once formed, which dissolves in the excess of benzoyle chloride; a drop of the latter, if now heated with potassa solution, dissolves readily, while the ether is not acted upon. The reaction is very evident if twenty or twenty-five c.c. of water are used containing only one per cent. of alcohol. But even with a few c.c. of water containing only one-thousandth of alcohol, the odor of the ether is still manifest.—*Répertoire de Pharm., Nov.,* 1871.

